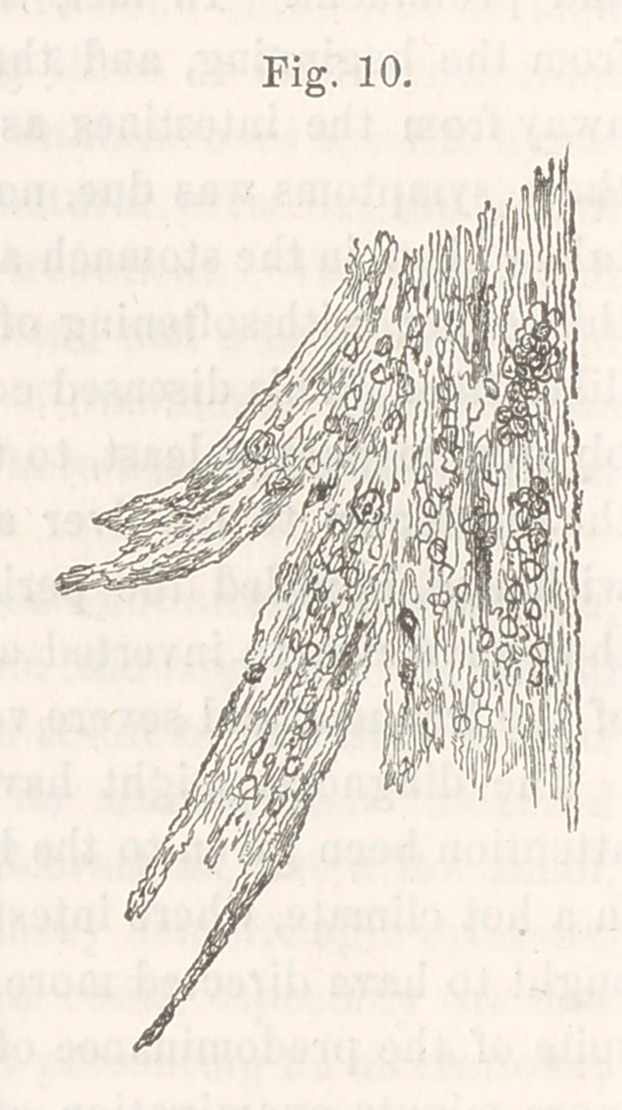# Proceedings of the Pathological Society of Philadelphia

**Published:** 1860-01

**Authors:** 


ART. VI.—*Proceedings of the Pathological Society of Philadelphia.*

WEDNESDAY EVENING, Sept. 28th, 1859.

The President, DR. LA ROCHE, in the Chair.

*Chronic Thickening of the Peritoneum, occasioning a Friction Sound.*—DR. HUTCHINSON remarked on this case as follows:—

Daniel Smith, aged 66 years, a native of Germany, a ragpicker, was admitted into the Pennsylvania Hospital August 12th. We could learn very little of his history; but he asserted most positively that he had been sick for only six weeks, and that, previously to that time, he had been able to follow his usual occupation. His son has since told us, that twenty years ago it was thought he had phthisis, but that he entirely recovered, and has since then enjoyed good health.

At the time of his admission he was exceedingly emaciated and pale. He stated that he had become so during his illness. His pulse was always over 100, and in the evening it frequently ran up as high as 120. His skin was harsh, dry, and hot. His tongue slightly coated and dryish. The percussion was abnormally clear over all parts of the chest, except between the scapulæ; on the left side it was slightly dull; on the right, more so. A mucous râle was heard at the lower part of the right lung; in all other parts the respiration was normal. There was no cough, excepting toward the close of his life. The abdomen was drawn in, but not irregularly nodulated, as it usually is in tubercular peritonitis; it was tender to the touch, and imparted a sensation of doughiness to the hand. When the hand was passed over the abdomen, so as to cause one surface of the peritoneum to rub against the other, a grating sound was heard, and on one occasion a full respiration was sufficient to produce it; grating was also felt distinctly by the fingers. Percussion over the region of the liver indicated rather a diminution of its size. The spleen was not enlarged. He had one or two passages from his bowels daily, which were small and whitish. The urine was natural. The treatment adopted was entirely of a supporting nature. He was removed from the hospital three days before death, and died September 25th.

An autopsy was made eight hours after death. The head was not examined. *Thorax.*—Both lungs were found bound to the parietes of the chest by old adhesions, the right completely so; they were studded with large irregular masses, which were found in some cases to inclose a cretaceous substance; in the neighborhood of these nodules, and in other parts of the lungs, there was a deposit of carbonaceous matter. A similar deposit had taken place in the bronchial glands. The only healthy portion of the lungs was the lower lobe of the left lung. The heart was of normal size, and presented no traces of disease, with the exception of a slight ossific degeneration of the aortic valves. *Abdomen.*—The peritoneum was found to be very much thickened, having in the median line a thickness of a quarter of an inch; on its surface were numerous minute elevations, about the size of a millet-seed. The whole of the small intestines were glued together. The liver was so adherent to the diaphragm that some force was necessary to separate it from its attachments. It was found somewhat smaller than natural, and had upon its surface bodies similar to those seen upon the peritoneum; the finger could readily be passed through its substance. The pancreas, stomach, and kidneys were healthy. There was but a small amount of effusion into the cavity of peritoneum. The deposit in the lungs, when submitted to microscopical examination, was believed to be tuberculous; its being cretaceous, and surrounded by a carbonaceous substance, gave rise to the opinion that it had been deposited twenty years before his death.

I may state, as bearing on the case, that Bennett, in his "Lectures on Clinical Medicine," says, "Nothing is more common than to see chronic tubercles surrounded by black pigmentary matter." Rokitansky also notices the association of black pigment with old tubercle.

---

WEDNESDAY EVENING, Oct. 12th, 1859.

Vice-President, DR. STILLÉ, in the Chair.

*Chronic Gastritis, Duodenitis, and Colitis.*—DR. J. F. MEIGS exhibited, through the Secretary, specimens of these disorders; accompanying them were these notes of the case:—

William C., aged 22, born in Ireland, a hackman in Philadelphia; had been in this country five years. Never sick in bed before last spring; but once, while in Ireland, had had a pain in the breast. Went to Cuba about March 1st, to work on the railroad from Havana to Matanzas; was taken sick with severe fever three days after arrival; was delirious, and very sore over stomach. He was cupped on breast and back of neck, and well purged and vomited. He was sick ten days before he went into the hospital, where he lay about two months; in the hospital he had chills and fever, and a little diarrhœa. Started for home about the end of May. During last half of passage was very sick; had severe diarrhœa for two weeks. Landed at Philadelphia 19th June, where he lay at a boarding-house, very sick. He had chills, and was cured; then seized again, and entered the Pennsylvania Hospital July 14th; was treated in hospital; went out much better, after two weeks, and did pretty well until Thursday, August 18th, when, late in the day, after dinner, he threw up his meal and a *large quantity* of dark blood; entered hospital again Monday, August 22d.

*Condition on admission.*—Very pallid and unhealthy looking; just the aspect of chronic intermittent; not emaciated, but somewhat thinned; quite able to walk about; no fever; no pain, except in epigastric region, where there was decided tenderness, without tumor. He had at this time very slight diarrhœa: ordered him quinine and iron, diet of arrow-root and milk, and to lie abed. But he grew gradually worse, ejected almost everything at times from the stomach, and then would pass two and three days without vomiting. The diarrhœa was moderate at first; stools thin, like gruel, containing flocculent, feculent matter, floating in liquid; color yellowish and brownish; no mucus noticed; no tenesmus; no griping; no blood at any time seen in the stools; at no time indeed was there the characteristic slimy and bloody discharge of dysentery. Tongue generally clean; never much furred; smooth, soft, and moist, until toward the end. It was never red, glazed, nor shining; but smooth, and at times rather polished; no aphthæ nor exudation. At no time had he any violent fever, yet there was at times a slight febrile irritation; no sweatings. Nothing, either dietetic or medicinal, exerted any positive control over the symptoms. Milk, mixed with lime-water, and containing small quantities of brandy, was tried after the arrow-root gruel with milk had failed. Beef tea, chicken soup, meat of chicken, mutton chop, all failed in turn; toward the last, yolk of egg in wine, with small quantities of beef essence, suited best. After the quinine and iron, he was treated with creosote, with bismuth and chalk, with kino and opium, and laudanum enemata, with tannin, were used. When he had been in the house two weeks, he became decidedly, though never deeply, jaundiced. This subsided, and disappeared about two weeks before his death; still, the urine never contained bile, and the stools were never clay colored. When he entered there was decided enlargement of spleen; it could be felt, jutting three or four inches from beneath the left margin of the thorax, and the dullness over the left hypochondriac region was augmented beyond its natural area, but the size of the organ diminished after about two weeks treatment. We also found unusually strong pulsation of the abdominal aorta; and a hard body could be felt across the aorta, just above and to left of umbilicus. Dullness was observed under the right margin of thorax. There was also a well-marked systolic mitral murmur.

Autopsy.—The lungs were healthy. The heart not altered in structure, even the mitral valves being quite healthy, and showing that the mitral murmur, heard during life, must have been the result of the anæmic state of the blood. Stomach very much dilated; its mucous coat thickened and softened, and deep in color; no large ulcerations, but a few small, superficial ones, such as are called by Rokitansky hemorrhagic erosions. Duodenum very considerably dilated, and all its coats, especially the mucous, thickened, and the latter also softened, but presenting no ulcerations; the lower extremity of this intestine was found to be fastened to the edge of the liver and to the gall-bladder, by quite strong adhesions. Pancreas enlarged and hardened, but not scirrhous, nor exhibiting any appearance of recent inflammation. Spleen natural. Liver decidedly enlarged, with its lobular structure extremely well marked; it presented the appearance called nutmeg. Kidneys presented nothing remarkable. Small intestines healthy. Large intestines extensively and most seriously diseased. The cæcum showed an advanced condition of inflammation and ulceration.

The ascending and transverse colon exhibited the same appearances, in a somewhat less advanced degree. The descending colon and the rectum presented a mucous membrane deeply inflamed and ulcerated; the color dark, deep red, with very numerous ulcerations, while between the ulcerations portions of mucous membrane retained their natural structure, except that they were deep red in color, and thickened. The appearances here seen were such as Habershon has described; large portions of the mucous membrane being lost, the remaining portions looking like small polypoid formations.

The most interesting feature of this case was the singular latency of the colitic disease, so far as any dysenteric symptoms went. The patient had neither pain in the intestine, soreness to the touch, mucous, or bloody stools. There was, I am sure, at no time, though the stools were repeatedly and carefully examined, any appearance of mucus, blood, or pus; nor a complaint of pain, nor tenesmus, or straining. There was severe diarrhœa toward the last, but no dysentery, and the gastric symptoms, as exhibited by hæmatemesis, and almost constant vomiting, were most severe and prominent. In fact, the gastric symptoms predominated so much from the beginning, and throughout the case, that attention was drawn away from the intestines as the chief seat of disease. The severity of these symptoms was due, no doubt, to the extensive changes which had taken place in the stomach and duodenum, both of which organs exhibited thickening, with softening of the mucous membrane, and very considerable dilatation. This diseased condition, especially the dilatation, were probably due, in part at least, to the adhesions which fastened the lower end of the duodenum to the liver and gall-bladder. These adhesions interfered with and impeded the peristaltic movement of these two viscera, and thus gave rise to inverted action, and assisted greatly in the production of the frequent and severe vomiting.

The diagnosis might have been in all probability made, had proper attention been given to the history of the case. The origin of the attack in a hot climate, where intestinal diseases and complications are frequent, ought to have directed more careful observation to the large intestines, in spite of the predominance of the gastric symptoms, when, perhaps, a still more minute examination of the stools might have revealed deposits of pus, or shreds of false membrane.

DR. GROSS did not think it possible that a mucous surface as diseased as the one on the table should not have secreted pus.

DR. KELLER believed that the patient had been seized at the onset of the attack with dysentery, and not with fever, as reported.

*Report of Committee on a Plastic Cast of the Bronchial Tubes.*—The committee to whom the specimen of plastic exudation from the bronchi, exhibited by Dr. S. D. Gross at the last meeting but one of the Society, was referred for examination, beg to submit the following report:—

Examined microscopically, the structure was found to consist of very fine fibres, arranged nearly parallel to one another, and having a slightly wavy course, (ordinary areolar tissue.) Nuclei were abundantly distributed along the bands so formed, and some free nuclei were also present.

A small number of non-ciliated epithelial cells of the squamous variety, probably from the bronchial tubes, were also observed.

The committee compared this case with the analogous ones collated by Dr. Peacock, in the Proceedings of the London Pathological Society, vol. v., and with those published by the same gentleman, in the London Medical Times and Gazette for 1854; and they regard this specimen as quite remarkable for its size and perfectness.

Fig. 9.

Fig. 10.

The drawings accompanying the report show the cast as it appeared to the naked eye; and its fibres, dotted with nuclei, as exhibited by the microscope.

JOHN H. PACKARD, } *Committee.*

WM. KELLER, }

Dr. Packard then presented a paper "On the Pyogenic or Suppurative Diathesis," the reading of which was deferred until the next meeting.

---

Wednesday Evening, Oct. 26th, 1859.

The President, Dr. Stillé, in the Chair.

Dr. Packard read the following paper:—

ON THE PYOGENIC OR SUPPURATIVE DIATHESIS.

The above term has been preferred to those which have from time to time been applied to the affection now to be discussed, as being least open to the charge of prejudging the matters at issue. The name by which it is most commonly designated, *pyæmia,* expresses a theory not universally received; and so also does that of *purulent absorption,* or *resorption of pus,* both of which have found advocates. All these titles are significant of the changes in opinion which have taken place in regard to the character of the disease. And we shall find that these changes have been due, not to the discovery of fresh facts, but to the different constructions placed upon observations entirely analogous to one another. For here, as in all other arguments upon matters not within the limits of mere abstract truth, the great point is to arrive at the exact relations of cause and effect. Owing, however, to the complicated conditions to be considered, this inquiry is peculiarly difficult in physiology, and still more so in pathology.

In order to study to the best advantage the subject now before us, we shall in the first place survey the facts at command, and then the views and theories hitherto based upon them; lastly, we shall seek to define the conclusions derivable from this examination.

The essential pathological condition to be investigated is, the occurrence of suppurative inflammation, generally at several points, in some organ or organs distant from the seat of a local inflammatory disorder; the former phenomenon being generally observed in one or more of the viscera, and without any apparent local cause, while the latter is apt to subside more or less completely, as if by a shifting or metastasis of the inflammation to a new locality.

Thus after an amputation, the discharge of pus from the stump is very apt to cease if this condition is set up; if it occurs in a puerperal woman, the lochial flow is diminished or altogether suspended. But such is not always the case; sometimes the suppuration still continues at its original seat, or is changed only in quality. However this may be, the pus deposited secondarily is apt to be quite healthy in its character; its formation is very rapid.

Phlebitis is a very common, if not an invariable element of the disorder; it commences at the part primarily affected, and extends to a greater or less distance along the vein, usually, however, throughout its whole length. It manifests itself in the same way as when idiopathic.

Now the secondary inflammations of the viscera may stop short of suppuration, simply because death takes place at too early a period for this stage of the local process. But they inevitably terminate in it, and not in resolution, if life lasts long enough; the lymph effused is not plastic, but *pyogenic* in the true sense of that word. Another peculiarity consists in their circumscribed character; they do not tend, however near to one another, to inosculate. Each focus, like a boil on the skin, has its own centre from first to last. Of all the organs, the lungs are most commonly attacked in this way, and next perhaps the liver; the brain sometimes, but less frequently, and still more seldom the spleen. Cases are upon record in which abscesses were found in the kidneys, and one or two in which the heart was the seat of irregular purulent formations.

The general symptoms are those of irritation with debility. In some cases, there are no evidences of serious derangement of the system, until the chill which is apt to precede the febrile movement. But sometimes this symptom is only noticed after several days of depression of spirits, malaise, and gastric derangement; or even after signs of pulmonary or hepatic congestion have declared themselves decidedly. The chill may be of any grade and duration, and may or may not recur. The fever which ensues either has from its commencement, or takes on subsequently, the typhoid type; the pulse becomes excessively rapid, the face dusky, the abdomen meteoric; the teeth become covered with sordes, and the tongue dry and brown, or else smooth and red. Delirium is almost always, perhaps invariably present; it may be of the low and muttering kind, but sometimes there is wild excitement. Toward the last there is frequently vomiting of dark-brown matter, probably altered bile, which is ejected without much effort, often apparently without consciousness. Death seems to result from general exhaustion, much as in other cases of a typhoid type.

Two symptoms are sometimes observed in these cases, in a marked degree, without any condition being discoverable to account for them. At a very early stage of the disorder, even before the chill comes on, there may be acute pain, like that of rheumatism, about one or more of the joints; the wrist is often its seat. This pain may be premonitory of an abscess at the part, but is by no means invariably so. At a later period there may be an extremely marked jaundice, which is occasionally due to suppuration in the liver, but does not certainly indicate it.

The following notes of cases may serve to illustrate the sketch now given:—

CASE I.—*Purulent infection following a blow upon the toe; death on the eighth day. Autopsy.*

William Jones, aged 14, born in Philadelphia, was employed as errand-boy about a market-house. On Friday, June 11th, 1859, he struck the great toe of his left foot against a brick.

The skin was not broken, and he did not complain much until the following day, when the foot became intensely painful, red, and swollen. On the 13th he became delirious.

Leeches were freely applied to the foot, and a wash, composed probably of lead-water and laudanum.

On the 16th he was seen by my friend Dr. Dunton, to whom I am indebted for these notes. His left foot was of a livid-red color, hard, and much swollen, but cool. The limb above was hot, and there were patches of redness here and there in the course of the internal saphena vein; another patch of redness existed at the outer side of the right foot. He was delirious, with a hot skin, and a rapid and compressible pulse; his tongue was moist, but thickly coated; he had some cough, and frequent green liquid stools.

Lead-water and laudanum were applied locally, and he was ordered a mixture of Spts. Mindereri and Tr. Opii Camph., with a diet of milk and beef tea.

On the 17th, his left foot had assumed a dark-purplish color, as had also the spot on the right foot. A thick hard cord could be felt in the course of the internal saphena vein. He was much more delirious, and more prostrate; his diarrhœa was checked. He complained of pain in the left side when moved.

Additional stimulus was ordered, and Quiniæ Sulph. gr. j., every two hours.

He, however, sank steadily, and died very quietly at 10½ P.M. By the kindness of Dr. Dunton I assisted at the autopsy, seventeen hours after death. Body muscular and well developed; rigor mortis well pronounced. Both feet swollen and congested; the swelling extended up to the groin of the left side. Hypostatic congestion over nearly the whole of the posterior or under surface of the body and limbs.

On taking off the skull-cap, the dura mater was found somewhat adherent to its inner surface. Some bloody serum flowed out when the ventricles were laid open. The brain-substance was normally consistent, that of the left hemisphere somewhat congested.

*Thorax.*—Croupous lymph in both pleural cavities. The lungs presented, near their surfaces, foci of inflammation, of all sizes below that of a walnut; at the centres of a few of these suppuration seemed to be commencing. Heart healthy; normal clots in both cavities.

*Abdomen.*—Viscera and veins entirely normal.

The *left leg* was very carefully examined in reference to the state of the veins. It was œdematous, and the internal saphena vein and its tributaries were distended with puruloid matter, their coats thickened and reddened. Here and there a very small vein was found filled with a red blood-clot; but, as a general rule, the distending substance was a yellowish matter, varying in consistence from that of pus to that of an ordinary clot. At the central portion of the vein was a clot about fifteen inches in length, tapering gradually at either extremity, and apparently breaking down into pus. At the point of entrance of the saphena vein into the femoral, a firm clot blocked up the passage completely. The femoral vein was perfectly healthy. A small collateral vein to the saphena, arising from it about two inches below its termination, and emptying into it again just at the latter point, was perfectly healthy, containing a red clot extending its whole length.

Just at the metatarso-phalangeal articulation of the left great toe was an abscess, laying bare the bone, but apparently not entering the joint.

CASE II.—*Secondary abscesses following amputation; death on the twenty-third day. Autopsy.*

This patient was a very muscular negro man, aged about 30. His right thigh was amputated by Dr. Norris, at the Pennsylvania Hospital, on the 24th of December, 1856; the operation was rendered necessary by a gun-shot fracture. A ligature was placed upon the vein.

After his death, the lungs were found full of small abscesses and foci of congestion; the heart very large, and the coronary veins distended; the liver very large, and universally congested; the spleen soft. On raising the pelvis, pus and blood flowed from the right iliac vein, apparently coming from both the femoral and the gluteal veins. During life, he had complained greatly of pain in the *left* shoulder-joint; but this was found to be perfectly healthy.

CASE III.—*Secondary abscesses following amputation; death on the twenty-seventh day. Autopsy.*

This patient was also a colored man, aged 35, whose feet had been frosted. His right leg was amputated January 21, 1857, by Dr. Peace, at the Pennsylvania Hospital. A great many ligatures were necessary, and one was applied to a vein.

After death, the lungs were found full of abscesses. The right femoral vein and its tributaries, except the saphena and the anterior tibial, were distended with pus. Although the skin and tissues were intensely yellow, the liver was perfectly healthy.

In this case, as well as in the last, the symptoms of purulent infection were manifested within twenty-four hours after the ligature came away from the vein, which was in this case found to be patulous; its condition in the other was not noted.

The left popliteal vein was also full of pus; but it must be remembered that the left foot was also frost-bitten. Circumstances prevented a very thorough examination being made.

CASE IV.—*Abscess of the Liver following amputation; death on the twenty-first day. Autopsy.*

Michael McGorry, aged 21, had his wrist so badly crushed and luxated as to require amputation, which was done by Dr. Neill, at the Pennsylvania Hospital, August 3, 1856. Symptoms of purulent infection declared themselves in a few days, and he died on the 24th, three weeks after the operation.

A post-mortem examination showed a purulent deposit in the liver while the lungs were entirely healthy. The brain was congested, and there was some serous effusion in the ventricles. One kidney was very large and pale.

Dr. Hall* reported to this Society, in October last, a case of purulent infection following upon amputation of the upper arm. At the autopsy, pus was found in the veins of the stump; sero-purulent liquid existed in considerable quantity in the pleuræ and pericardium; the lungs were studded with small abscesses; the spleen was of a bright-red color, and at its lower portion presented a mass of gangrene the size of an egg; liver healthy.

CASE V.—*Purulent infection (?) following amputation; death on the sixteenth day. Autopsy.*

George Stouffer, aged about 30, an American farmer, was admitted into the Pennsylvania Hospital, July 25th, 1855, suffering from chronic disease of the left knee. For this his thigh was amputated by Dr. Pancoast, on the 1st of September. On the fourth day the stump was attacked with erythema. This was checked in a day or two; but on the eighth day each forearm became the seat of similar inflammation. He now began to sink; he had several attacks of almost suffocating dyspnœa, from inability to throw off the mucus which collected constantly in his throat; and chemosis occurred to an extreme degree in both conjunctivæ. The stump shrank up very remarkably, and he died on the sixteenth day. He had had no marked chill at any time.

At the autopsy, tuberculous deposits were found in each lung, but no other visceral disease. On the back of each forearm, just above the wrist, was a subcutaneous abscess full of healthy pus.

---

* North Am. Med.-Chir. Review, Jan., 1859, p. 96, or Proceedings of Path. Society of Philadelphia, p. 147 of the current volume.

CASE VI.—*Purulent infection following amputation; death on the tenth day.*

Sarah Ann Bush, aged 45, was admitted into the Pennsylvania Hospital on January 7th, 1856, for compound comminuted fracture of both bones of the left leg, close to the ankle. She was in the habit, according to her statement, of taking f℥iss of laudanum daily.

Amputation was performed by Dr. Peace, the same evening. Of course, it was absolutely necessary to administer opium in large doses, on account of her previous habits.

No bad symptom occurred until the seventh day, when she complained of great pain in the right wrist. The next evening she had a chill, and on the next day (the ninth after the operation) she became jaundiced. Stimuli were now given freely.

On the 17th, (the tenth day after the operation,) I was called to her early in the morning; found her with an anxious countenance and a tympanitic abdomen; the jaundice was extreme. Ordered an enema of turpentine; the other stimuli to be continued. At ten o'clock I was again called, and found her dying; vomiting up immense quantities of bile and undigested food, which flowed both from the nose and mouth, without any straining or effort.

Her friends would not allow any post-mortem examination to be made.

The foregoing cases are presented as exemplifying the course of the disease of which we have now to study the pathology. They are not all so complete as they might, or, perhaps, should have been—but it is one design of this paper to call attention to the subject, and to induce the pathologists of this country to give it more consideration than it has hitherto obtained; and the least full of the above records is still not without value in regard to some special point.

It is well known that abscesses in various parts of the body are apt to follow certain affections, such, for instance, as enteric fever; these local sequelæ usually occur at the surface, and seem to be a result of some specific influence of the preceding disease upon the blood. Of the extent and importance to which they may attain, the following case will be illustrative:—

CASE VII.—*Abscesses following enteric fever; recovery.*

Israel Braddock, aged about 35, a colored porter, came under my care in the latter part of September, 1858. His symptoms were at first such as might have indicated bilious-remittent, but in a few days they became clearly those of enteric fever. My friend Dr. Dunton attended him in consultation with me; he was for several days extremely ill, but at length showed signs of amendment.

During the seventh week of his disorder, he one day complained of pain in the region of the left scapula. Upon examination, a very distinct fluctuation was perceptible at the spot; and an incision gave exit to an enormous quantity of thin but healthy pus.

Soon after this, he was removed to the Pennsylvania Hospital, where he remained until spring; several other abscesses of large size formed about his thighs and in his back, and it was for a long time doubtful whether he could survive the drain thus established upon his system. He, however, eventually regained his full health and strength, and was discharged cured.

We sometimes see abscesses form, after injuries of the extremities, at more or less remote points. These may be due simply to the inflammation of a lymphatic gland in direct connection with the part affected, although even then it becomes a question *how* the sympathetic action is induced; or they may be, to all appearance, independent of any such anatomical relation. Thus I have seen an enormous formation of pus, so large that it could hardly be ascribed to glandular inflammation, in the groin of a man whose toe had been crushed between two wheels; there was no free suppuration at the seat of injury.

But there are cases in which suppuration occurs secondarily, after injuries which would seem inadequate to such a result; of these the following case affords an example:—

CASE VIII.—*Purulent deposits following a slight injury of the knee; hectic fever, and death.*

George Bradshaw, aged about 45, was brought to the Pennsylvania Hospital, January 4th, 1856, having the day before slipped on the ice and strained his right knee. He had spent the night at a house close by where the accident happened, having been unable to walk; but there was no evidence of any injury to the joint. The thigh was slightly swollen, and somewhat erythematous.

He was a very large, stout Englishman, and had lived heartily, although, according to his statement, not intemperately.

It became evident, a few days after his admission, that an abscess was forming at the outer side of the thigh. This was freely opened, and exit given to a large quantity of pus. After this, several other abscesses formed, and were opened at different points along the thigh; then one occurred behind the knee; and, lastly, openings had to be made on each side of the leg. The orifices remained patulous, shreds of dead areolar tissue came away, and the discharge was very profuse. His strength began to give way, in spite of stimuli and nourishment; hectic fever set in at about the 1st of March, and on the 4th of April he died.

No autopsy could be obtained; but there had not been, from first to last, the slightest indication of any other lesion than these abscesses in the intermuscular areolar tissue.

Let us now inquire into the opinions of authors upon the subject with the elucidation of which we are especially engaged at present. It would not be worth while to notice at length the speculations of those who wrote before the time of Harvey; but the following passage, from the introduction to the Second Book of Paré's Works, shows that that great surgeon had observed and reasoned upon these so-called metastatic abscesses:—

"Besides, also, which furthermore argues a great putrifaction of humors, many had Abscesses in parts opposite to their wounds, as in the left knee, when as the right shoulder was wounded; in the left arme, when as the right Leg was hurt. Which I remember befell the King of *Navarre*, the Duke of *Nevers*, the Lord *Rendan*, and divers others. For all men had nature so overcharged with abundance of vicious humors, that if it expelled not part thereof by impostumes to the habite of the body, it certainly otherwise disposed of it among the inner parts of the body; for, in dissecting dead bodies, wee observed that the Spleene, Liver, Lungs, and other Bowells were purulent, and hence it was that the patients, by reason of vapours sent from them to the heart, were troubled with continuall feavers."

Now, there are various ways in which the occurrence of secondary suppurations may be accounted for.

1. An explanation has been sought in the idea that when the head is injured by a blow, a fall, or a concussion of any kind, a like concussion is sustained by the liver or lungs, which may thus become inflamed, and suppurate. This view was defended chiefly, I believe, by Richerand; it is entirely set aside by the fact that in many cases no concussion whatever is sustained; as, for instance, when an amputation is the starting-point of the disorder.

2. The solidists, Desault, Bichat, and others, ascribed the phenomena in question to *sympathy;* a term which, although convenient, and sometimes even indispensable, is too vague to be of much value here.

3. The admixture of pus with the circulating blood, as a cause of metastatic abscesses, was I believe first mentioned by Boerhaave. His idea was that the pus became changed in quality by confinement, and that it eroded the extremities of the veins and lymphatics, so as to gain access to their interior. This idea of *pyæmia* has always obtained more or less favor among pathologists, but its acceptance does not close the discussion of the subject, since the greatest difference of opinion may still exist both as to the source and the mode of action of the pus. It is with these questions that we have now to deal.

But the very first inquiry to be made is, "What effect has pus upon blood when mixed with it?" To obtain an answer to this it is unnecessary to go beyond the experiments of Sédillot, who repeatedly injected large quantities of pus into the veins of dogs, without any marked effect; for if pus had the coagulating agency ascribed to it by some, or the absolutely poisonous action ascribed to it by others, the results must have been by no means negative. It is well known, also, that many writers maintain the possibility of the removal of even large deposits of pus by absorption; although it is difficult to perceive how any positive evidence of such an occurrence can be obtained. Very few surgeons, probably, will agree with Cruveilhier, when he goes so far as to say:—

"The absorption of pus, like that of all secreted liquids, is so much a part of nature's plan that I do not believe there is a single instance of the termination of inflammation without it."

On physiological grounds, however, the idea of any true absorption of pus may be rejected. Absorption is the entrance of any substance in the liquid form through a membrane, without any solution of continuity in the latter. Now, as Sédillot remarks, pus without corpuscles is not pus at all, and it is physically impossible for a pus-corpuscle to pass through the sound wall of a vein or of a lymphatic; while the experiments of this author have shown that the liquor puris is destitute of any injurious effect when introduced into the blood.

Pus, however, like all other animal matters, is subject to decomposition; and authors have asserted that its injurious effects are due to its putrescent state when taken into the blood. Two facts have a certain amount of weight against this idea; in the first place, metastatic abscesses do not always follow the injection of other putrid matters into the veins of animals, although typhoid symptoms and death are induced; in the second place, all the phenomena of pyæmia may occur in a case where the primary suppuration retains its laudable character. Hence it is necessary to seek the rationale of purulent infection elsewhere than in the mere admixture of pus with blood, or in the putrid condition of the former liquid.

A mechanical theory which has been advocated by some high authorities, is embodied in the word *metastasis; metastatic* abscesses being supposed to be caused by a transference of pus-corpuscles mixed with the blood to an interior organ, where they act as obstructions in the capillaries. Cruveilhier, having abandoned his original idea of the tubercular character of these secondary deposits, adopted the one just mentioned, and became, perhaps, its chief exponent. He introduced mercury in substance into the femur of a dog; some time afterwards he found it in the animal's lungs, each globule of mercury forming the centre of a small abscess. When injected into the mesenteric vein, a like effect was produced in the liver. Hence he inferred that pus was similarly disposed of, and defended the following proposition :—

"Any foreign substance introduced bodily into the venous system, when its elimination by the emunctories is impossible, determines visceral abscesses exactly similar to such as supervene upon wounds and surgical operations, and these abscesses are the result of capillary phlebitis of those viscera."

The same view was defended by Mr. Simon, of London, as late as 1850. But it is entirely set aside by some considerations, the chief of which are, that it does not account for secondary abscesses in the areolar tissue, or in joints; that an organic cell would not, by lodging in a capillary, cause such an extensive interruption of the collateral circulation, whatever might occur if a foreign body were to lodge in like manner; and that such a theory leaves unexplained the rapidity, gravity, and peculiarity of the general symptoms.

Looking, however, at the clinical aspects of the subject, what evidence is there of the introduction of pus into the circulating blood in these cases? Here we must inquire whether pus can be detected, either by the naked eye or with the microscope, in the blood; for if so a great step may be gained. In the second of the cases already detailed, blood mixed with pus was seen to flow from the iliac vein. Piorry asserts that pus may even be detected in the blood drawn from pyæmic patients in venesection. But in the case just alluded to, the mixture of the two liquids was probably simply mechanical, and caused at the moment; Legallois, Dance, Sédillot, and others affirm that under other circumstances they could not be distinguished from one another.

Nor can we attain to any greater certainty by microscopic examination. Lebert's opinion, that there exists a visible difference between the pus-corpuscle and the colorless corpuscle of the blood, is entirely opposed to that of Virchow, Donné, and I believe of most other authorities on the subject. That the two cells are identical is by no means assumed, but only that they cannot be distinguished from one another with any certainty by their visible properties.

But admitting the *possibility* of the entrance of pus into the blood, does it *probably* take place in these cases?

To obtain an answer to this question we must first ascertain the source from which the pus might be derived. The idea of a simple transference the pus from the part primarily affected has already been set aside, as well as that of a true absorption of it; but may not a small portion be taken up by an open vein, by what some writers have called a process of aspiration? Some cases would seem to favor this latter supposition, which is, however, entirely overthrown by a single instance related by Bennett, in which the primary disorder was acute articular rheumatism.

Breschet, in 1817, first advanced the idea that a suppurative inflammation of the veins might furnish the pus by which the mass of the circulating blood was contaminated; and the convenience of this theory has recommended it in the eyes of many. Cruveilhier, indeed, goes so far as to say "*La phlébite domine toute la pathologie.*" Tessier, on the contrary, says that the word phlebitis has been a veritable nursery of error; affirming that whenever a vein is inflamed and suppurates, its communication with the rest of the circulatory apparatus is cut off, either by adhesive inflammation at its cardiac extremity, or by a plug of coagulated blood at the same point. Of the truth of this statement, the first of the cases I have detailed affords evidence; it is only one of a great many which are upon record. Moreover, in Bennett's case, before alluded to, all the symptoms of the so-called pyæmia occurred, but the veins were found, at the post-mortem examination, to be quite healthy. Bérard speaks of analogous cases. Tessier affirms positively that phlebitis is present in only five or six out of every ten cases of purulent infection, and argues strongly against the possibility of any relation of cause and effect between the two morbid conditions. In this opinion he is supported by Velpeau.

Phlebitis, on the other hand, does not by any means invariably cause metastatic abscesses. Duplay gives a series of eight cases in which the umbilical vein was inflamed, in children from two to twelve days old; peritonitis and icterus were constant symptoms, but there were no visceral abscesses, which he says do not ever occur in this form of disorder. In more advanced life, cases are not unfrequently met with in which the veins are inflamed, but no purulent infection ensues.

From the foregoing review of the opinions which have been advanced concerning the disorder in question, we see that neither transference nor absorption of pus can be accepted as the primary morbid phenomenon; and that phlebitis, although apparently offering a more substantial basis for theory, does not really stand the test of rigid examination. Authors have, therefore, been obliged to allege some additional cause for the symptoms observed, besides the mere admixture of pus with the blood. But this idea has so firmly seated itself in the minds of many, that they still allow it fundamental importance; thus Lebert assigns three periods to the disease: that of the formation of pus within the veins; that of the mixture of pus with the mass of the blood; and that of the pyogenic diathesis. Others again, having in view the analogy between the disorder in question and those caused by certain animal contagions—the plague, glanders, etc., speak of the blood as *poisoned;* but they still either express or imply the idea that it is the pus which constitutes the poison.

From what has been said, I think it will appear that there are defects in all the theories and hypotheses as yet put forth, in explanation of the occurrence of multiple abscesses after local injuries and surgical operations. Peculiar difficulties exist in tracing the phenomena of the disorder under consideration, so that the temptation is very strong to accept the most convenient doctrine, perhaps without sufficiently rigid scrutiny. It is probably for this reason that so many writers have defended views which a calm and impartial examination shows to be untenable.

Where, then, are pathologists of the present day to stand in regard to this most important affection? I beg to offer some considerations which may tend to a solution of the problem, although it may well be doubted whether its entire comprehension is within the power of the human mind.

In the first place, it is useless to attempt the task of accounting for the localization of such morbid actions as are due to constitutional causes. Thus the variolous poison gives rise to multiple abscesses in the skin; we know that such is the case, but we cannot go back of this fact. Nor can any one explain the mode in which the disease is communicated, when there is no actual contact between the person affected with it and the person receiving it. And so, also, that more strictly contagious disease, the glanders, is transmitted probably in the form of a material poison; but how or why that poison gives rise to the peculiar symptoms of the disease, no one can tell. It may be by a self-propagating power possessed by the poison, or by a catalytic influence upon the blood at large.

Again, after that clearly specific disease, enteric or typhoid fever, it is well known that abscesses are apt to occur here and there, without any local cause being assignable for them.

The simpler and much more frequent instance of boils and carbuncles may be adduced, as showing the effect of some inappreciable agent, (called, for the sake of convenience, a condition of the system,) in giving rise to suppurations apparently quite arbitrary in their choice of position. Every one knows that these may occur in persons seemingly in perfect health; and yet a tonic and stimulating regimen will often act most beneficially in such cases, showing that there was in reality a lowered and not an exalted condition of the vital powers.

Hence the process of suppuration, which may so commonly be traced to a local cause, may also be set up under the influence of agents modifying the constitution of the individual affected; whether these agents be considered as producing their effects through the nervous system, or by a taint impressed upon the circulating blood, or by altering the vital forces in amount or direction. In some cases, as has been seen, the action of a poison must be acknowledged; in others there is a state of the system at large, with which the local phenomena are obviously in connection; in others again such a connection can be so clearly inferred as to admit of no reasonable doubt, although it cannot be demonstrated.

Now comparing these facts with those which have been brought forward with respect to secondary suppuration after surgical operations, injuries, or local inflammations, they may afford a clue to the real pathology of this affection. For here, as in the other cases mentioned—pustular skin diseases, abscesses after typhoid fever, boils, and carbuncles—there exists a constitutional state, to which the name of *purulent diathesis* has been applied. This name was first proposed, I believe, by Tessier; I would suggest the word *pyogenic* as more expressive of the idea intended.

The *purulent*, or *pyogenic diathesis*, then, I would consider as present wherever there is a marked tendency to suppurative inflammation; as is often seen in hospital residents, sailors on board ship, and others who are exposed to depressing influences. By the pathologists of the present day, inflammation is perhaps universally regarded as attended with an actual diminution of the vital powers of the part, and is known to occur more readily in persons whose health is impaired. Hence, a tendency to inflammation, and to its termination in suppuration, may be set up by the influence of any cause of such impairment; very often this agency operates for a length of time without exciting any suspicion of its existence. An individual may be thus weakened, without losing the appearance of vigorous health. But under the additional depression incident upon an operation, or upon any serious disorder of the system, the diathesis may show itself by the development of a general typhoid state, cropping out here and there, also, in the shape of local suppurations. Such may be supposed to have been the order of things in Dr. Bennett's case, where the primary affection was acute articular rheumatism.

It may, however, be objected, that the diminution of the suppuration at the seat of injury or operation, or of the lochial discharge, when the parturient uterus is the starting-point, is opposed to the view stated. But this diminution does not always occur, and when it does, it may be accounted for by the transference of the inflammatory action to other points.

Again, it may be asked how the frequent existence of phlebitis, in these cases, is to be explained. That this is not the essence of the disorder, or even an invariable element of it, has already been argued. The inflammation of a circumscribed extent of any vein or veins may be looked upon, I think, as simply a coincidence, especially when the extreme readiness with which it takes place is borne in mind.

As to the experiments on animals, in which the phenomena of the affection we have been discussing were produced by the injection of putrid pus into the veins, they only show that this is one way in which the pyogenic diathesis may be set up. That it is not the only way, is shown by the many cases met with in which the pus of a stump or of a wound is perfectly healthy until the constitutional symptoms have declared themselves.

Lastly, it may be objected that the view I have advanced leaves some important points unexplained.

Thus it offers no account of the manner in which the suppurative action is localized, or of its limitation to certain circumscribed spots; and it substitutes a pathology which may seem vague and far-fetched, for one which is clear and easy of comprehension. But, as regards the first two points, this disease is only added to a very large list of those whose localization is altogether unaccountable, as well as the peculiarities of their phenomena. Who can pretend to explain why herpes zona never passes the median line of the body, or why lepra and psoriasis are so apt to attack the knees and elbows? Who can explain why vaccination causes a sore of a certain form, running a certain course? And as to the accusation of vagueness, it is set aside by the fact, that upon this theory the secondary suppurations which occur after injuries, operations, etc., are placed in the same category with those to which they are allied, instead of being left to stand by themselves, anomalies in pathology.

In view of the great frequency and fatality of secondary suppuration, it is surely surprising that the subject has attracted so little attention in this country. The idea which I have sought to defend—that such abscesses, occurring after injuries or operations, are due to a *pyogenic diathesis*, this being a state of system not without analogy under other circumstances—is, I think, a new one. If tenable, it may afford a sounder basis for prophylactic or curative measures than those which have preceded it; and in this way I hope it may be made practically useful.

A discussion then took place on the subject of pyæmia, in the course of which Dr. Woodward remarked, that while he recognized the truth of what Dr. Packard had said with regard to the extreme difficulty of determining the existence of pus in blood as seen under the microscope—owing to the impossibility of discriminating between pus-corpuscles and the proper white corpuscles of the blood—still, in a case where these corpuscles are abnormally abundant, we can at least say that one of two conditions must be present: either the patient, from whom the blood has been drawn, is suffering under leukæmia, or there is pus mixed with the blood. To determine this the symptoms and history of the case must be taken into account, and can leave but little doubt as to the nature of the disorder. Dr. Woodward also instanced cases illustrative of the rapidity with which pus might form, and spoke of the readiness with which, under certain conditions, it might enter into the circulation.

DR. PACKARD replied, that while he was fully sensible of the truth of all the remarks Dr. Woodward had offered, he did not think that the presence or absence of pus in the veins was of much practical importance as regards the disease in question, since it had been clearly shown that pus, when injected into the veins, does not produce pyæmia

DR. HENRY HARTSHORNE observed, that although the secretion of pus in organs distant from the seat of injury might often be owing to purely diathesic influences, without any distinct connection existing between the secreting centres, as suggested by Dr. Packard, still there were cases—and he instanced two as having fallen under his own observation—in which the neighboring lymphatics had become red and hard, first at the seat of injury, and in which this induration and redness had rapidly traveled along the course of the lymphatic vessels to a distant gland, which had suppurated. Such cases, he thought, must at least be allowed to be owing to continuous inflammation, if not to the direct conveyance of the pus from one suppurating centre to the other by means of the lymphatic vessel.

---

WEDNESDAY EVENING, NOV. 9th, 1859.

The President, DR. STILLÉ, in the Chair.

*Morbid Growths occupying the Ventricles of the Larynx; Death from Suffocation.*—DR. GROSS exhibited a larynx, in the ventricles of which were granulations. The specimen had been sent to him by Dr. H. W. Ducachet, with the following description:—

"The subject to whom the larynx formerly belonged was a mulatto man, of large frame, Felix by name, and aged about thirty-five at the time of his death. He was a coachman, living in the town of Arroyo, Porto Rico. I first saw him on the 25th of September, 1851, and his answers to the several questions I then put to him, though imperfect, will give a tolerable idea of his history before that time.

"About six years previous, while waiting for his master and family at a ball, he fell asleep on the box; it commenced to rain, and he was drenched to the skin, and contracted a severe cold, which confined him for some days to the house but not to the bed. He described his symptoms to me at that time as only those of a severe cold, with, however, a great deal of pain on swallowing. No physician was called to him then, his master not thinking him sick enough for it. He recovered, and resumed his duties at the end of a week; but from that time was never able to speak above a whisper. There was no cough when I first saw him, or afterwards, and his master assured me that the man never had had any. At the end of some months, the inability to speak loudly still continuing, medical aid was called in, but everything was tried without avail.

"When he presented himself to me he was apparently in perfect health, with a good appetite, no cough, and no pain; there was a slight hoarseness, and occasionally an 'uncomfortable sensation in the throat, which caused a desire to swallow.' I examined him thoroughly, but could discover nothing in his lungs indicative of disease; yet detected, on his taking a long breath, a hissing sound on expiration, which conveyed to my mind the impression of the existence of a stricture in the larynx, the exact position of which I was unable to determine satisfactorily to myself. I tried cauterizations internally with the nitrate of silver, and counter-irritation externally, besides many other remedies, too numerous to mention; but all without producing any amelioration of his symptoms, and finally abandoned the case, convinced in my own mind of the exceeding obscurity of the disorder.

"Once afterwards, in 1853, he applied to me, complaining, as he described it, of 'pain in his throat.' I ordered him a bran and mustard poultice, on going to bed, and heard no more of him until the 12th of December, 1854, when I was called to him about four o'clock in the morning. I found him dying; he expired about half an hour after I reached the house. His wife told me that he had had a slight cold for several days, and had gone to bed early, with great difficulty of breathing. She gave him a foot-bath, and applied the poultice of bran and mustard to his throat, which, as she said, had given him relief during former similar attacks; but, falling asleep herself, she knew nothing of his condition until about half-past three o'clock, when she was awakened by his struggles.

"The autopsy showed his lungs to be in a perfectly healthy condition; so were all the abdominal viscera. The larynx was filled with a thick, ropy mucus, which had so wound itself around the membranous band which completely filled up the tube, as to cause the patient to die asphyxiated.

"I will add to the description given, that words could be heard tolerably well at a distance of not over three feet from him. There was not, at any time after I first saw him until his death, any perceptible increase or diminution in the strength of his voice; it was, in every sense of the word, a whisper."

Dr. Gross looked upon this as a most remarkable case. The morbid growth he believed to be coagulable lymph, the result of inflammation, and not a true polypus.

Dr. Stillé thought the fact of the case having originated in cold pointed more toward a plastic exudation than to a polypoid growth.

Dr. Edward Hartshorne stated, that in a case of laryngeal polypus which had come under his observation at the hospital, not only were the symptoms entirely different during life, but the post-mortem appearances were dissimilar. In the case to which he alluded the man had breathed for several years through a very small orifice, and had finally died suddenly from suffocation, with exactly such symptoms as attend a case of membranous croup. After death the larynx was found almost entirely closed, the orifice being scarcely large enough to admit a small probe.

*Miliary Tubercle in the Lung; Tubercle of the Liver; Psoas Abscess; in a man seventy-six years of age.*—DR. KELLER, in exhibiting these specimens, said :—

Mr. Ertel, seventy-six years of age, emigrated about fifteen years ago from Germany, and has since that time been employed in one of the great chemical factories in this city, where he was occupied with the manufacture of different chemical preparations. He had consulted me several times during past years for various ailments, but on the twenty-third of June of this year, for the first time, for a cold abscess of the size of the fist, extending from the fourth to the sixth rib, on the right side, near to the sternal edge. I directed him to paint it with tincture of iodine, which he did for a short time; but not thinking that any treatment could relieve such an old and debilitated person, I soon lost sight of him. I was again called to see him on the sixteenth of October, when I found him in bed, very weak, and scarcely able to recognize me. His hands and feet were œdematous, his pulse at the wrist not perceptible. This condition had not changed on the next day. On the nineteenth day the œdema had disappeared; the pulse could be felt, but it was very feeble and irregular; the respiration was more difficult. He died during the following night. The post-mortem was performed on the twenty-second, by Dr. Packard, whose report I give :—

Autopsy, October 22.—The abscess being laid open, was found to contain several ounces of pus, and a good deal of semi-solid, cheesy matter, forming a sort of inclosure for it. The fourth rib was thickened about three inches on its sternal end; it was eroded, and could be felt at the bottom of the cavity. The sternum, with part of the aorta, was similarly affected, and fragile, like the denuded part of the rib.

Opposite this point, the lung was strongly adherent to the parietes of the chest, by means of bands of organized lymph. Toward its base, on its posterior surface, this lung was attached to the wall by a very thick and dense mass of the same character.

The right lung was congested with blood, and had miliary tubercles diffused everywhere through its substance; but it still swam in water. The other lung was adherent to the chest-walls by most of its surface; it was studded with miliary tubercles, and pieces of it sank instantly when dropped into water.

The two layers of pericardium were everywhere adherent, except on the under surface of the heart. The heart's substance was increased in bulk, but softened, and was found, under the microscope, to be in a state of fatty degeneration. On the left side both sets of valves were thickened, and ossific deposit had occurred in the subserous tissue forming the base of the valves. The coronary arteries were ossified to some extent.

The liver was enlarged, fatty, and anæmic, with points of tuberculous deposit here and there in its substance; one mass of this nature was of about the size of a chestnut, and seated at the under surface. Spleen soft and deep colored. Kidneys somewhat pale and flabby; on the surface of one of them was a whitish deposit, about as large as a grain of wheat, presenting no very marked microscopic characters.

The mesenteric glands were very much enlarged, hard, and converted into a white, cheesy material, exactly like that mentioned as found at the under surface of the liver.

In the right axilla three or four glands were enlarged, one to about the size of a large chestnut, the others in less degrees. These all exhibited the same change into a cheese-like material.

All these deposits, being placed under the microscope, were found to consist of small, irregularly shaped, but generally ovoidal cells, containing bright, oil-like particles, and many of them also a nucleus of comparatively large size, with one or two nucleoli.

In the psoas muscle, on the left side, there was an accurately limited abscess, laying bare a small portion of the fifth lumbar vertebra. The psoas muscle was diminished in volume, somewhat greenish in color, and of less than normal consistence. Under the microscope, it seemed to be breaking down, its elements were less distinct than usual, and appeared to be, as it were, dissolved.

*Bright's Disease; Waxy Kidney.*—DR. HARLAN exhibited, for Dr. Reed, a specimen of this disease, and read an account of the case:—

Frederic Smith, tailor, aged twenty, native of Germany, was admitted into the Pennsylvania Hospital, October 20th, 1859. He stated that, when about ten years old, he had had an attack of general dropsy, which yielded to rest and treatment in about three weeks, and that after his arrival in this country he had an attack which lasted about the same time, but was not followed by dropsy; his health had been good up to the commencement of the month. Since that time he has had dropsy, which he cannot satisfactorily account for; he believes it to be owing to a profuse perspiration which was suddenly checked. When examined on admission, he presented the waxy-white appearance peculiar to albuminuria. There was some effusion into the cavity of peritoneum; the urine was scanty and highly albuminous; the pulse small but regular; the tongue pale; the bowels costive and not easily operated upon; the appetite good. He was ordered to take diuretics; a diet nutritious but not stimulating, and hot vapor baths every other night.

On the seventh, Dr. Meigs showed the patient to the class. He notes: "Patient has passed about three pints of urine in the twenty-four hours. It is amber colored, specific gravity 1006 to 1008, and highly albuminous. The dropsy extends up around the loins and to the walls of the abdomen; the effusion into the abdomen is slight; there is also slight effusion into the right pleural sac. The dullness over the heart extends about two inches above the nipple, along the edge of sternum; the apex beat is feeble yet distinct. It contains small, waxy-looking casts, epithelial cells in abundance, and some blood. Heart probably pushed up by the diaphragm, as the abdomen is very considerably distended by flatus and fæcal accumulation; if any ascites, it is very moderate."

On the twenty-sixth of September, when I examined the case, the general condition of the patient had not varied much from the description given.

On the third of October, elaterium (one-eighth gr.) was ordered. This produced nausea and vomiting. As it had the desired effect upon the bowels (four to six stools in twenty-four hours) it was persevered in. The dose was repeated on the fifth, fifteenth, and eighteenth. About the fourth of October a peculiar venous congestion or stagnation was noticed in the epigastric vessels, and, to a slight extent, upon the thighs. This condition gradually increased until about ten days before death, when, simultaneously with the decrease of the œdema, the vessels were relieved.

About the seventeenth, the patient was suddenly attacked at night with torturing pains and swellings in the abdomen and thighs, which gradually extended down the limbs to the feet. It was impossible to touch his body without producing pain; and the weight of the bedclothes had to be supported by hoops. This condition, which seemed to resemble both phlebitis and inflammation of the lymphatics, was treated with Dover's powders, liniments, and bandages, and passed off in five or six days.

Following closely upon this was a decided loss of appetite. The bitart. potas. and infus. juniperi were put aside, and acetate of potas. and comp. spts. juniperi ordered instead. This was about the twenty-fourth of October. The œdematous condition daily diminished, and the abdomen, by the thirtieth of October, had assumed its natural dimensions. The swelling of the limbs was also partly lessened. The patient now refused solid food, and took milk-punch and soups; tonics did no good. On the thirtieth he was decidedly weaker, and brandy and water was ordered; after this he gradually became more and more prostrated, and died comatose on the thirtieth of November. The last six days of his life he complained of very severe pains in his bowels, and was kept but partially quiet by full doses of opium. Ten days before death a diarrhœa set in which proved intractable, and doubtlessly hastened the fatal result

Notes of post-mortem twelve hours after death. Brain not examined. Lungs have small tubercular deposits scattered through both; at top of left lung, posteriorly, is a small abscess containing a quantity of tubercular matter. The liver and spleen are normal or nearly so. Stomach and duodenum healthy. Pancreas has what seem to be tubercular matter in it; studding the internal surface of the ileum are a number of ulcerations, tubercular in character. Some of the mesenteric glands are enlarged, and, in some four or five, a hard deposit, like cast-off lime, is found. The kidneys are large and waxy. A subsequent examination showed that there was no abnormal amount of fatty degeneration, and the whole case will perhaps most properly be regarded as the "waxy degeneration" of Dr. Todd. The amount of urine passed did not vary much through the treatment of case—from five to eight pints in the twenty-four hours.

In this case there certainly was not enough of tubercular disease to cause death. Patient died comatose, and for some hours before death his symptoms showed plainly the presence of urea in the blood. Perhaps his was one of those cases mentioned by Dr. Todd, where, although the normal amount of urine was secreted, yet the proper elimination of its elements did not take place.

During the whole progress of the disorder there seemed to be but little aggravation of general symptoms. Cough and bronchitis same throughout; pulse varied but slightly, 86 to 106; no headache; appetite generally good; amount and character of urine passed much the same; bowels operated on by medicines until the last two weeks of life. The post-mortem showed the lymphatic glands, which lay along the course of the iliac veins, very much enlarged and hardened. The pressure which these glands exert on these vessels may, perhaps, account for the great venous congestion of the external vessels of the abdomen and thighs. Certainly the amount of fluid in the peritoneum could not account for it. Though this explanation does not seem very plausible, yet it is an effort to account for one of the most singular and interesting complications of the case.

*Fracture of the Anatomical Neck of the Humerus.*—Exhibited by DR. LENOX HODGE. A few days ago, a muscular German, forty-three years of age, fell, about twenty-five feet, from a tree. He died the same afternoon, from injuries to his body and pelvis. The humerus gave every indication of being fractured through its anatomical neck. So it proved. The fracture extended from the tubercles above downward and inward along the neck, and below passed forward through the capsular ligament into the head. This was the principal line of fracture; at various points of its course several small fragments were split off.

*Ossific Deposit in the Pleura.*—DR. HODGE continued. Upon opening the thorax of the German, whose humerus has just been exhibited, we found extensive pleuritic adhesions, especially on the left side. We removed the lungs, and on the posterior surface of the left, between the pulmonary and costal pleuræ, we saw what appeared to be a portion of a rib. But, upon examination, we could detect no rib to be absent, or broken, or denuded of its periosteum. The deposit is of the consistence and appearance of bone, lies along the fissure between the lobes of the left lung, and is four and a quarter inches long, by one-half inch to three-quarter inch broad, and about one-eighth of an inch thick. It is closely adherent to both layers of the pleura, and, at its anterior extremity, seems to project through the costal pleura, at least into the subserous areolar tissue. A small nodule of a similar deposit was found among the adhesions at the summit of the same lung.

Feeling the importance of ascertaining positively the nature of this deposit, I respectfully submit it to the action of the Society. Bone is often said to be deposited in the pleura after inflammations; but those cases in which a microscopical examination has been made, the deposits, though called ossific, osseous, or osteid,* have generally and perhaps always proved not to be true bone.

The specimen was referred, for minute examination, to a committee.

*Tumor of Breast.*—DR. LENOX HODGE next exhibited a mammary tumor. About three years ago, an abscess followed a contusion of the left breast of a married woman, twenty-seven years of age, and the mother of several children. The abscess opened, discharged, and the part returned, apparently, to a healthy state. One year afterwards, without any known cause, an abscess formed in the axilla of the same side. This also went through its stages and the parts returned to their normal condition. Last spring the patient gave birth to a child, which lived six days. She had

* Transactions of the Pathological Society of London; "Ossification of Pleura, by Dr. Bristowe, vol. ii. p. 35; "Ossific Mass deposited in the Pleura," by Dr. Hyde Salter, vol. v. p. 35; "Osteoid of the Femur, with Osteoid Growth in the Lungs, Omentum, and Diaphragm," by Dr. Hillier, M.B., vol. vi., 317. The Osteophytes, reported by Dr. J. Varise, (Archives Générales de Médecine, tome xxi. 4th S., pp. 320 and 448,) were not deposited in the pleura, but on the ribs by the periosteum.

plenty of milk; and, after the death of the child, her breasts dried up, without any untoward symptom. Then, however, she noticed, for the first time, a small tumor on her left breast, near the nipple, and felt shooting pains through it, especially during changes in the weather. There was no increase of size till the middle of September, when her husband died. Since then, it has increased rapidly, and become much more painful, so that, during the last two weeks, she has had almost constant pains, by night and by day. The tumor was removed this morning by Dr. Norris. It is large and hard, and with a marked retraction of the nipple. The skin is thickened, especially in points, forming hard nodules. Except, perhaps, its size, it presents the characteristics of scirrhus of the breast. A small tumor, of apparently the same nature, was found above this, and nearer the axilla. It also was removed. The axilla itself was unaffected.

Dr. Packard stated that he had made a microscopical examination of a minute portion of the tumor, and that he had found it to consist of gland-tissue, tubes lined with epithelium, and imbedded in a fibrous stroma. He was desirous of studying it more thoroughly.

Dr. Woodward thought the tumor resembled the lardaceous cancer described by Velpeau.

## Figures and Tables

**Fig. 9. f1:**
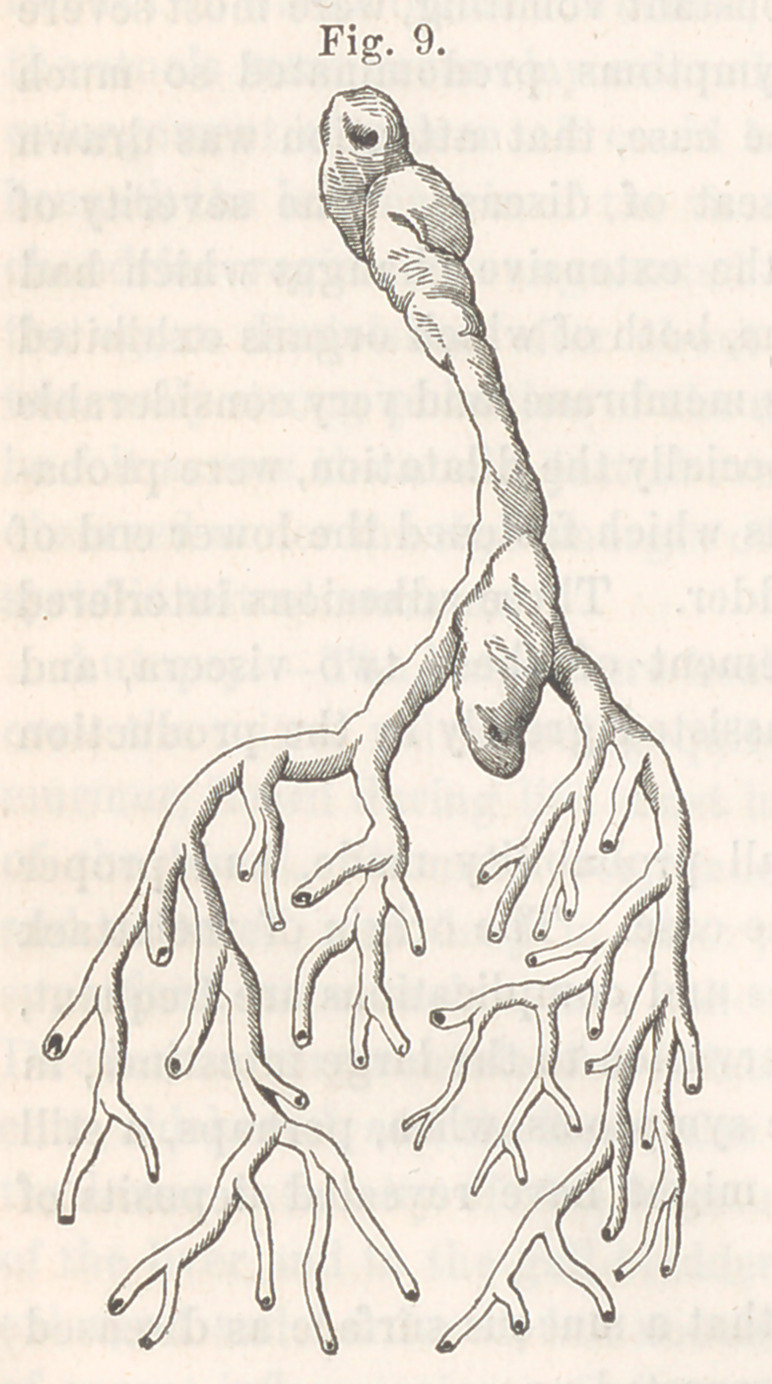


**Fig. 10. f2:**